# The Worst-Case Weighted Multi-Objective Game with an Application to Supply Chain Competitions

**DOI:** 10.1371/journal.pone.0147341

**Published:** 2016-01-28

**Authors:** Shaojian Qu, Ying Ji

**Affiliations:** 1 Business School, University of Shanghai for Science and Technology, Shanghai, P.R. China; 2 The Logistics Institute-Asia Pacific, National University of Singapore, Singapore, Singapore; Nankai University, CHINA

## Abstract

In this paper, we propose a worst-case weighted approach to the multi-objective *n*-person non-zero sum game model where each player has more than one competing objective. Our “worst-case weighted multi-objective game” model supposes that each player has a set of weights to its objectives and wishes to minimize its maximum weighted sum objectives where the maximization is with respect to the set of weights. This new model gives rise to a new Pareto Nash equilibrium concept, which we call “robust-weighted Nash equilibrium”. We prove that the robust-weighted Nash equilibria are guaranteed to exist even when the weight sets are unbounded. For the worst-case weighted multi-objective game with the weight sets of players all given as polytope, we show that a robust-weighted Nash equilibrium can be obtained by solving a mathematical program with equilibrium constraints (MPEC). For an application, we illustrate the usefulness of the worst-case weighted multi-objective game to a supply chain risk management problem under demand uncertainty. By the comparison with the existed weighted approach, we show that our method is more robust and can be more efficiently used for the real-world applications.

## 1 Introduction

Multi-objective game is a generalization of the scalar criterion game and is used to model situations where two or more decision makers, called players, take actions by considering their individual multiple objectives. The study for multi-objective game can be dated back to Blackwell’s work where the author considers the zero-sum games with multi-objective payoffs [1]. Since then much attention has been attracted to the game models with multiple payoffs [2–4]. One of the reasons is that multi-objective models can be better applied to real-world situations [2].

In this paper, we consider *n*-person worst-case weighted multi-objective games where each player has two or more objectives and is ambiguous about the weights to the objectives. More specifically, we focus on finite multi-objective games where the weights of objectives are uncertain and assumed to belong to an convex and compact set. In our model, we suppose that all players are risk-averse and a player uses the robust optimization approach to manage the weight uncertainty, assuming that other players are robust optimizers as well. Note that the robust optimization approach is not concerning game data or the other players’ strategies, but concerning the weights to the objectives.

In multi-objective games, since each player needs to consider several competing objectives and it is not possible to simultaneously optimize all objectives, so a commonly accepted approach for coping with this setting is the weighted approach by assigning a nonnegative weight by considering the importance of the corresponding objective function. And then a player can make a decision by optimizing a weighted sum objectives by assuming that other players also make their decision by optimizing their own weighted sum objectives [2, 3, 5]. A weighted Nash equilibrium point can be obtained if each player makes his decision by optimizing his weighted sum function. However the current weighted approach has several shortcomings. As shown in applications, the weights are not known in advance and the player has to choose them. Ambiguity often exists in the choice of the weights to objectives, as it is not easy to decide relative weight for each objective. In addition, as shown in multi-objective optimization in the literature that relative weights given by the same decision-maker may rely on the elicitation methods [6, 7]. Therefore, it is necessary to provide a new approach to cope with these issues.

Hence, our motivation to utilize a worst-case weighted approach is that it provides an alternative way to deal with the weights ambiguity. Furthermore, if each player in multi-objective games chooses the worst-case weighted approach, then we show that there is at least a robust weighted Nash equilibrium which further guarantees the existence of the Pareto Nash equilibrium. The computation for such an equilibrium, with the choice of polytope weight set for each player, is reformulated as a solution to a mathematical program with equilibrium constraints (MPEC) which can be solved by the existed methods (for example the sequential quadratic programming (SQP) methods).

A concrete example that could receive the benefit of our model takes place in a supply chain which contains multiple risk-averse retailers who compete in quantities of a set of products to satisfy the uncertain consumer demand. By considering the wholesale price each retailer chooses its wholesale market order quantity for each of the products in order to maximize the mean of its profit and minimize the standard deviation of its profit simultaneously. Each retailer has more than one objectives to be considered and the corresponding robust weighted Nash equilibrium can be obtained by utilizing the proposed method in this paper.

### 1.1 Summary of Contributions

We feel that the primary contributions of this paper are:

- We propose a worst-case weighted approach for multi-objective *n*-person non-zero sum games, extending the notion of robust weighted multi-objective optimization models to multi-objective games. Our work can also be seen as an extension of the robust one-shot scalar games.
- We prove the existence of a robust weighted Nash equilibrium even when the weight sets chosen by the players are unbounded. Our proof extends the existence proof for the weighted Nash equilibrium in multi-objective games [2, 5].
- We then show that a robust weighted Nash equilibrium point can be calculated by solving a MPEC when the weight sets chosen by the players are polytopes. We note that the MPEC has been extensively studied and can be solved by the sequential quadratic programming (SQP) method [8, 9].
- We demonstrate the usefulness of worst-case weighted multi-objective games in a multi-objective competition problem within supply chain.

### 1.2 Literature Review

Our work relates several streams in the literature in robust optimization, multi-objective optimization and game theory. Robust optimization approach is an effective methodology to deal with the optimization problem contaminated by uncertainties [10, 11]. However, there are few studies for utilizing robust optimization approach to analyze the multi-objective optimization. Some technology from robust optimization has been successfully extended to the multi-objective optimization setting, but computational and theoretical challenges remain (see, for example, [12–16]). Hu and Mehrotra present a robust and stochastically weighted approach to the multiobjecitve optimization by considering inconsistency and ambiguity in relative weights given to objectives [12]. Cromvik and Lindroth introduce a new definition of robustness for multi-objective optimization based on some utility function [16]. Soares et al. present a definition of the worst-case Pareto optimum and discuss its applications in design optimization [13, 14].

This paper is also related to the incomplete information game theory area, the robust game formulation has been extensively utilized to lower the performance variation under the parameters uncertainty [17–19]. In the robust game setting, it is supposed that the parameters are not known exactly, but they can be any number of a known set without distributional information and a robust game reformulation is utilized to cope with such parameter uncertainty. In these papers, the authors prove the existence of a robust equilibrium point and show that the computation for such a point can be cast as some computationally tractable problem under the standard assumptions about the uncertain parameters. However, to the best of our knowledge there is no research about utilizing the robust optimization approach to the multi-objective games.

Since “many real-life decision-making situations involve a choice between options which will influence the degree of achievement of each of several objectives” [20], so there are many researchers study multi-objective optimization. White is one of them who has done a great job in the theory, computation and applications in the multi-objective optimization stream [21, 22]. Recently as an extension to multi-objective optimization, the games with multi-objective payoff functions attract people’s interest. The first work can be dated back to Blackwell’s zero-sum games with multi-objective payoffs [1]. Then, Shapley introduces the concept of equilibrium points in games with vector payoffs [23]. Since then much attention has been attracted to the game models with multiple payoffs [2–4]. However, the studies mainly focus on the existence proof of a Pareto Nash equilibrium and the improvement for the solution concepts [2, 5, 24–28]. For example, by fixed point theorem and other techniques, Wang [2], and Yu and Yuan [5] prove the existence of Pareto equilibria. Yuan and Tarafdar study the existence of weighted Nash equilibria and Pareto equilibira for multi-objective games with noncompact strategic sets [28]. Borm et al. present the existence theorem of the Pareto Nash equilibrium for two person multicritera games [26]. Borm, van Megen and Tijs [24], Zhao [25], and Radijef and Fahem [27] study the improvement for the solution concepts. The well-posedness for multi-objective games is also studied. For example, Peng and Wu [4] present a generalized Tykhonov well-posedness concept; Morgan [29] discusses the parametrically well-posedness for multi-objective games. There are few studies to discuss the applications and to present the numerical algorithm for obtaining an equilibrium point in multi-objective game.

### 1.3 Outline of the Paper

This introductory section is concluded with notations which will be used in this paper. In section 2, we review some basic concepts in multi-objective game theory and then introduce a new concept of robust weighted Nash equilibrium. We show that a robust weighted Nash equilibrium is also a weighted Nash equilibrium and then is also a Pareto Nash equilibrium. In section 3, we prove the existence of a robust weighted Nash equilibrium by utilizing Kakutani’s fixed point theorem. We also prove that there is at least one robust weighted Nash equilibrium point even when the weight sets chosen by the players are unbounded. In section 4, we show that when the weight sets are given as polytopes, the robust weighted Nash equilibrium can be cast as a MPEC which can be efficiently solved by SQP methods. In section 5, we show the efficiency of our approach by an example for analyzing a multi-objective competition problem within supply chain. Finally, we conclude this paper in section 6.

### 1.4 Notation

Throughout this paper we use the following notations. *R* is the set of real numbers; *R*_+_ denotes the set of non-negative real numbers and *R*_++_ is the set of strictly positive real numbers; Let

$$\begin{array}{c} R^{m}=\underset{m}{\underset{︸}{R\times \cdots \times R}}\;;R_{+}^{m}=\underset{m}{\underset{︸}{R_{+}\times \cdots \times R_{+}}}\;;R_{++}^{m}=\underset{m}{\underset{︸}{R_{++}\times \cdots \times R_{++}}}\;; \end{array}$$

For any *u*, *v* ∈ *R*^*m*^, denote

$$\begin{array}{ccc} & & v\geq u\;\left(resp.\;v\leq u\right)⟺v-u\in R_{+}^{m}\;\left(resp.\;u-v\in R_{+}^{m}\right)\\ & & ⟺v_{j}-u_{j}\geq 0,\;j\in I\;\left(resp.\;v_{j}-u_{j}\leq 0,\;j\in I\right); \end{array}$$

$$\begin{array}{ccc} & & u≻v\;\left(resp.\;v≺u\right)⟺v-u\in R_{++}^{m}\;\left(resp.\;u-v\in R_{++}^{m}\right)\\ & & ⟺v_{j}-u_{j}>0,\;j\in I\;\left(resp.\;v_{j}-u_{j}<0,\;j\in I\right); \end{array}$$

$$\begin{array}{ccc} & & v≽u\;\left(resp.\;v≼u\right)⟺v-u\in R_{+}^{m}\;and\;u\neq v\left(resp.\;u-v\in R_{+}^{m}\;and\;u\neq v\right)\\ & & ⟺v_{j}-u_{j}\geq 0,\;j\in I\;and\;u\neq v\;\left(resp.\;v_{j}-u_{j}\leq 0,\;j\in I\;and\;u\neq v\right); \end{array}$$

where *I*: = {1, ⋯, *m*} is the index set; Given any vector function *h*: *R*^*n*^ → *R*^*m*^, by *h* ∈ *C*^1^(*R*^*n*^, *R*^*m*^), we indicate that *h* is a continuously differentiable function from *R*^*n*^ to *R*^*m*^ and we use ∇*h*(*x*)∈*R*^*n* × *m*^ to denote the gradient of the function *h* at *x*; For *x*: = (*x*^1^, ⋯, *x*^*n*^), we define (*x*^−*i*^, *u*^*i*^) as the vector with all components same as that in *x* except *i*th component being *u*^*i*^, that is (*x*^−*i*^, *u*^*i*^): = (*x*^1^, ⋯, *x*^*i* − 1^, *u*^*i*^, *x*^*i* + 1^, ⋯, *x*^*n*^).

## 2 The Robust Weighted Multi-objective Game

In this paper, we consider the following multi-objective game with finite players in the normal form, *MG*: = (*N*, {*S*_*i*_}_*i* ∈ *N*_, {*F*^*i*^}_*i* ∈ *N*_), here *N*: = {1, ⋯, *n*} is a finite set of players, $S_{i}\subset R^{a_{i}}$ is the set of actions for player *i* and *F*^*i*^ is the multi-objective payoff function of player *i* which is a mapping from the Cartesian product $S:=\prod_{i\in N}S_{i}$ into $R^{b_{i}}$ (since this paper considers the multi-objective game, so without loss any generality throughout this paper we assume that each player *i* has more than one objectives in his payoff function, that is *b*_*i*_ ≥ 2). Specifically, given *x*: = (*x*^1^, ⋯, *x*^*n*^)∈*S* to be played, then each player *i* has his multi-objective payoff function as $F^{i}\left(x\right):=\left(f_{1}^{i}\left(x\right),\cdots ,f_{b_{i}}^{i}\left(x\right)\right)$ which means that each player *i* has more than one competing objectives.

Since there are more than one objectives for each player in the above multi-objective game, so a comprehensively accepted strategy for each player is the (weak) Pareto optimal strategy which is a concept borrowed from multi-objective optimization and can be defined as follows (see [2–4]).

**Definition 2.1** *For any player* *i*, *i* ∈ *N*, *whose strategy* *x*^*i*^ ∈ *S*_*i*_ *to the other player’s strategies* $x^{-i}\in S_{-i}:=\prod_{j\in N,j\neq i}S_{j}$ *is called a Pareto optimal strategy (resp. a weak Pareto optimal strategy) if* *x*^*i*^ *is a Pareto optimal strategy (resp. a weak Pareto optimal strategy) respectively to the following multi-objective optimization*,

$$\begin{array}{c} \min_{x^{i}\in S_{i}}\;\;F^{i}\left(x\right), \end{array}$$ (1)

*that is* *x*^*i*^ ∈ *S*_*i*_ *is called a Pareto optimal strategy (resp. a weak Pareto optimal strategy) of player* *i* *to the other player’s strategies* *x*^−*i*^ ∈ *S*_−*i*_ *if there is no strategy* *u*^*i*^ ∈ *S*_*i*_ *such that*

$$\begin{array}{c} F^{i}\left(x^{-i},x^{i}\right)≼F^{i}\left(x^{-i},u^{i}\right)\;\;\left(resp.\;\;F^{i}\left(x^{-i},x^{i}\right)≺F^{i}\left(x^{-i},u^{i}\right)\right). \end{array}$$

With the above definition, we can give the Pareto (resp. weak Pareto) equilibrium concept for multi-objective game which can be expressed as a mixed strategy where each player’s strategy is a Pareto optimal strategy (resp. a weak Pareto optimal strategy) to the other player’s strategies.

**Definition 2.2** *A mixed strategy* *x* ∈ *S* *is called a Pareto (resp. weak Pareto) equilibrium for MG if for each player* *i*, *x*^*i*^ ∈ *S*_*i*_ *is a Pareto optimal strategy (resp. a weak Pareto optimal strategy) against* *x*.

The above Pareto (resp. weak Pareto) equilibria concept has been extensively used in multi-objective game which is closely related with another concept-the weighted Nash equilibrium.

**Definition 2.3** *A mixed strategy* *x* ∈ *S* *is called a weighted Nash equilibrium with given weight combination* *w*: = (*w*^1^, ⋯, *w*^*n*^) $\left(w^{i}\in R_{+}^{b_{i}}\right)$ *of MG if for each player* *i*, 0 ≼ *w*^*i*^ *and* *x*^*i*^ *is the optimum to the following optimization problem*

$$\begin{array}{c} \left(P^{i}\left(w^{i}\right)\right)\;\;\min_{u^{i}\in S_{i}}{\left(w^{i}\right)}^{T}F^{i}\left(x^{-i},u^{i}\right). \end{array}$$

*In special case with* ∥*w*^*i*^∥_1_ = 1, ∀*i* ∈ *N*, *the mixed strategy* *x* ∈ *S* *is called a normalized weighted Nash equilibrium*.

The above definition means that a normalized weighted Nash equilibrium is a corresponding weighted Nash equilibrium. However in the following lemma we show that a weighted Nash equilibrium is also a corresponding normalized weighted Nash equilibrium. Therefore in the rest of this paper, we always assume that the weighted Nash equilibrium is the normalized weighted Nash equilibrium, that is we always suppose that the given weight *w*: = (*w*^1^, ⋯, *w*^*n*^) satisfies $w^{i}\in W_{r}^{i}:=\left\{w^{i}\in R_{+}^{b_{i}}|\;∥w^{i}{∥}_{1}=1\right\}$, ∀*i* ∈ *N*.

**Lemma 2.4** *If a mixed strategy* *x* ∈ *S* *is a weighted Nash equilibrium of MG with given weight combination* *w* = (*w*^1^, ⋯, *w*^*n*^) *and* 0 ≼ *w*^*i*^, ∀*i* ∈ *N*, *then* *x* *is also a normalized weighted Nash equilibrium of MG with some weight combination* $\bar{w}:=\left({\bar{w}}^{1},\cdots ,{\bar{w}}^{n}\right)$, *where* ${\bar{w}}^{i}\in W_{r}^{i}$, ∀*i* ∈ *N*.

**Proof.**The assertion follows directly from both definition 2.3 and the conclusion that for any *i* ∈ *N*, optimization problems (*P*^*i*^(*w*^*i*^)) and $\left(P^{i}\left({\bar{w}}^{i}:=\frac{w^{i}}{∥w^{i}{∥}_{1}}\right)\right)$ have the same optimal solutions.

In the MG literature, Definition 2.3 is one of the most often used concepts and many papers discuss it’s relationship to the Pareto equilibrium. If *x* ∈ *S* is a weighted Nash equilibrium of MG with given weight combination *w* = (*w*_1_, ⋯, *w*_*n*_) and 0 ≼ *w*_*i*_, ∀*i* ∈ *N* (resp. 0 ≺ *w*_*i*_, ∀*i* ∈ *N*), then *x* ∈ *S* is also a weak Pareto equilibrium (resp. Pareto equilibrium) to MG. However, the weighted approach is also criticized for several shortcomings. For example, as shown in applications, the weights are not known in advance and the modeler or decision-maker has to choose them while it is often difficult to make a reasonable decision for choosing appropriate weight for each objective, since in many situations there may be ambiguity in the weights provided by the decision-maker. Therefore, there is a need for providing a novel methodology to cope with these issues which is based on a worst-case approach.

In our paper, we suppose that each player *i* has a set *W*^*i*^ of weights for his objectives, then we can give a robust weighted Nash equilibrium concept for MG.

**Definition 2.5** *Given a close set* $W^{i}\subset W_{r}^{i}$, *i* ∈ *N*, *then a mixed strategy* *x* ∈ *S* *is called a robust weighted Nash equilibrium of MG if for each player* *i*, *x*^*i*^ *is the optimum to the following robust optimization problem*

$$\begin{array}{c} \left(RoP\left(W^{i}\right)\right)\;\;\min_{u^{i}\in S_{i}}\max_{w^{i}\in W^{i}}{\left(w^{i}\right)}^{T}F^{i}\left(x^{-i},u^{i}\right). \end{array}$$ (2)

For the simplicity, we denote by *RoP*(*W*^*i*^) for problem Eq (2) with given *W*^*i*^ and its optimal value (with the convention that *RoP*(*W*^*i*^) = +∞ if the problem is infeasible). For any *i* ∈ *N*, given two weight sets $W_{1}^{i}\subset W_{2}^{i}$, it is obvious that $\left(RoP\left(W_{1}^{i}\right)\right)\leq \left(RoP\left(W_{2}^{i}\right)\right)$. Furthermore, in this robust weighted Nash equilibrium concept, although we do not suppose the convexity of *W*^*i*^, ∀*i* ∈ *N*, actually the robust weighted Nash equilibrium concept of MG with closed weight sets combination $W:=\left(W^{1},\cdots ,W^{n}\right)\subset W_{r}:=\left(W_{r}^{1},\cdots ,W_{r}^{n}\right)$, is equivalent to the robust weighted Nash equilibrium concept of MG with closed weight sets *conv*(*W*): = (*conv*(*W*^1^), ⋯, *conv*(*W*^*n*^)) ⊂ *W*_*r*_, where *conv*(⋅) means the convex hull. This is actually equivalent to show for any player *i* and fixed *x*^−*i*^ ∈ *S*_−*i*_ the equivalence of the following two sets

$$\begin{array}{c} \chi_{i}=\left\{\left(\gamma ,x^{i}\right)\in R\times S_{i}|\;u^{i}\in S_{i},\;{\left(w^{i}\right)}^{T}F^{i}\left(x^{-i},u^{i}\right)\leq \gamma ,\;\forall w^{i}\in W^{i}\right\} \end{array}$$

and

$$\begin{array}{c} {\bar{\chi }}_{i}=\left\{\left(\gamma ,x^{i}\right)\in R\times S_{i}|\;u^{i}\in S_{i},\;{\left(w^{i}\right)}^{T}F^{i}\left(x^{-i},u^{i}\right)\leq \gamma ,\;\forall w^{i}\in conv\left(W^{i}\right)\right\}. \end{array}$$

It is easy from *W*^*i*^ ⊂ *conv*(*W*^*i*^) to see that for any $\left(\gamma ,x^{i}\right)\in {\bar{\chi }}_{i}$, (*w*^*i*^)^*T*^ *F*^*i*^(*x*^−*i*^, *u*^*i*^)≤*γ*, ∀*w*^*i*^ ∈ *W*^*i*^, that is (*γ*, *x*^*i*^)∈*χ*_*i*_, which means that ${\bar{\chi }}_{i}\subset \chi_{i}$. Next we are going to prove that $\chi_{i}\subset {\bar{\chi }}_{i}$. To this end, for any (*γ*, *x*^*i*^)∈*χ*_*i*_, next we will show $\left(\gamma ,x^{i}\right)\in {\bar{\chi }}_{i}$. For any *w*^*i*^ ∈ *conv*(*W*^*i*^), there exist *w*^*ik*^ ∈ *W*^*i*^, *k* = 1, ⋯, *κ*, such that $w^{i}=\sum_{k=1}^{\kappa }\lambda_{k}w^{ik}$, here λ_*k*_ ≥ 0(*k* = 1, ⋯, *κ*), and $\sum_{k=1}^{\kappa }\lambda_{k}=1$ for some *κ* ≤ *b*_*i*_ + 1. From (*γ*, *x*^*i*^)∈*χ*_*i*_, (*w*^*ik*^)^*T*^ *F*^*i*^(*x*^−*i*^, *x*^*i*^)≤*γ*, *k* = 1, ⋯, *κ*, which implies that

$$\begin{array}{c} {\left(w^{i}\right)}^{T}F^{i}\left(x^{-i},x^{i}\right)=\sum_{k=1}^{\kappa }\lambda_{k}{\left(w^{ik}\right)}^{T}F^{i}\left(x^{-i},x^{i}\right)\leq \gamma . \end{array}$$

This means that $\chi_{i}\subset {\bar{\chi }}_{i}$. Hence, $\chi_{i}={\bar{\chi }}_{i}$, ∀*i* ∈ *N*. Therefore, in the rest of this paper, we always assume that *W*^*i*^ is a closed and convex subset of $W_{r}^{i}$, ∀*i* ∈ *N*.

Different from the weighted Nash equilibrium concepts, the robust weighted Nash equilibrium proposed in this paper seeks to find an equilibrium solution that is robust, that is feasible for the worst-case weight within the family of weights. Wang shows that any normalized weighted Nash equilibrium of MG with weight given in $W_{r}^{i}$, ∀*i* ∈ *N*, then it is either a weak Pareto equilibrium or a Pareto equilibrium [2]. In the following proposition we show that the robust weighted Nash equilibrium of MG inherits the properties of normalized weighted Nash equilibria, that is any robust weighted Nash equilibrium of MG with weights set $W^{i}\subset W_{r}^{i}$, *i* ∈ *N* is either a weak Pareto equilibrium or a Pareto equilibrium.

**Theorem 2.6** *For any* *i* ∈ *N*, *given a close set* $W^{i}\subset W_{r}^{i}$, *if* x*: = (*x*^1*^, ⋯, *x*^n*^)∈*S* *is a robust weighted Nash equilibrium of MG with the combination* *W*: = (*W*^1^, ⋯, *W*^*n*^) *of weight sets, then we have the following results*,

1. x* is a weak Pareto Nash equilibrium of MG;
2. if for any player *i*, *i* ∈ *N*, all of its’ weights in *W*^*i*^ are all positive, then x* is a Pareto Nash equilibrium of MG;
3. if for any player *i*, *i* ∈ *N*, *x*^i*^ is the unique optimal solution of *RoP*(*W*^*i*^), then x* is a Pareto Nash equilibrium of MG.

**Proof.**The proof for this theorem directly follows from Theorem 2.2 of Hu and Mehrotra [12] and Definitions 2.1, 2.2 and 2.5.

Below we describe a class of multi-objective games that can be resolved by using robust weighted approach.

**Example 1** A set of *J* hospitals (the players) provide *I* types of healthcare services in order to minimize the social costs and minimize the dissatisfaction at the same time by anticipating the patient volume for services and the patients’ average strategic reactions to the prices of different services in each hospital. Hospital *j* (*j* = 1, ⋯, *J*) prices his *i*’s service (*i* = 1, ⋯, *I*) to minimize the social costs and minimize the dissatisfaction, assuming that the other hospitals keep their prices fixed, and anticipating the total patient volume as well as patients’ average strategic reactions to the prices of different services in each hospital. Mathematically, hospital *j* faces the following multi-objective decision problem,

$$\begin{array}{ccc} & & min\;\;\left(f_{j}^{1}\left(d_{j}\right),\;f_{j}^{2}\left(d_{j},p_{j}\right)\right)\\ & & s.t.\;\;0\leq d_{j}^{i}⊥E\left[u_{j}^{i}\left(\xi \right)-u_{j}^{i}\left(d_{j},p_{j}^{i},\xi \right)\right]\geq 0,\\ & & \;\;\;\;\;\;\sum_{j=1}^{J}d_{j}^{i}=D_{i},\;p_{j}^{i}\geq 0,\;\forall i,j, \end{array}$$ (3)

where $f_{j}^{1}\left(d_{j}\right):=\sum_{i=1}^{I}C_{j}^{i}\left(d_{j}^{i}\right)$ denotes the cost of *j*′ *s* hospital for providing I types healthcare services, $f_{j}^{2}\left(d_{j},p_{j}\right):=E[\sum_{i=1}^{I}c_{j}^{i}\left(d_{j}^{i},p_{j}^{i},\xi \right)d_{j}^{i}+{\hat{c}}_{j}\left(d_{j},\xi \right)d_{j}^{i}]$ denotes the dissatisfaction for *j*′ *s* hospital, $d_{j}:=\left(d_{j}^{1},\cdots ,d_{j}^{I}\right)$, $p_{j}:=\left(p_{j}^{1},\cdots ,p_{j}^{I}\right)$; $d_{j}^{i}$ and $p_{j}^{i}$ are the patient volume and the price for service *i* at hospital *j*; $\xi :\Omega \to R^{l}$ is a random variable defined on (Ω,F,P) with support *Ξ* to denote the social, medical and patient physical uncertainties; $c_{j}^{i}\left(d_{j}^{i},p_{j}^{i},\xi \right)$ and ${\hat{c}}_{j}\left(d_{j},\xi \right)$ denote the individual patients’ dissatisfaction level to the treatment of service *i* and the dissatisfaction level to hospital *j*′ *s*; $u_{j}^{i}\left(\xi \right)$ is patients’ utility for service *i* at hospital *j*; $u_{j}^{i}\left(d_{j},p_{j}^{i},\xi \right)$ is the individual patients’ utility function of receiving service *i* at hospital *j*.

If each hospital has a set of weights $W^{j}\subset W_{r}^{j}:=\left\{w^{j}\in R_{+}^{2}|\;∥w^{j}{∥}_{1}=1\right\}$, *j* = 1, ⋯, *J*, then a robust weighted equilibrium can be obtained if each hospital solves the following robust weighted problem, *j* = 1, ⋯, *J*

$$\begin{array}{ccc} & & min\max_{w^{j}\in W^{j}}\left(w_{1}^{i}f_{j}^{1}\left(d_{j}\right)+w_{2}^{j}f_{j}^{2}\left(d_{j},p_{j}\right)\right)\\ & & s.t.\;\;0\leq d_{j}^{i}⊥E\left[u_{j}^{i}\left(\xi \right)-u_{j}^{i}\left(d_{j},p_{j}^{i},\xi \right)\right]\geq 0,\\ & & \;\;\;\;\;\;\sum_{j=1}^{J}d_{j}^{i}=D_{i},\;p_{j}^{i}\geq 0,\;\forall i,j. \end{array}$$ (4)

An important equilibrium concept is the *ex post* equilibrium arising from Harsayi’s Bayesian scalar game model with incomplete information. It follows from this concept, we give the following equilibrium concepts named as *ex post* weighted equilibrium for MG. With all possible realizations of the weight within the family of weights, a tuple of strategies is said to be an ex post weighted equilibrium if each player’s strategy is a best weighted response to the other player’s weighted strategies. This definition is rigorously stated as follows.

**Definition 2.7** *A mixed strategy* (*x*^1^, ⋯, *x*^*N*^)∈*S* *is an ex post weighted equilibrium for MG with the combination* *W*: = (*W*^1^, ⋯, *W*^*n*^) *of weight sets if for any* *i* ∈ *N*, *x*^*i*^ *is the optimal solution of the following problem*,

$$\begin{array}{c} \min_{u^{i}\in S_{i}}{\left(w^{i}\right)}^{T}F^{i}\left(x^{-i},u^{i}\right),\;\;\forall w^{i}\in W^{i}. \end{array}$$

The above definition implies that if (*x*^1^, ⋯, *x*^*n*^)∈*S* is an *ex post* weighted equilibrium of MG, then it must be a weighted Nash equilibrium for any weights choosing from *W*. Note that this condition is quite strong, since it is easy to prove that every *ex post* weighted equilibrium of MG is a weighted Nash equilibrium with the corresponding weight from the combination of weight set. Furthermore, the following lemma describes the relationship of the *ex post* weight ed equilibrium and our robust weighted Nash equilibrium.

**Lemma 2.8** *Given a close set* $W^{i}\subset R_{+}^{b_{i}}$, *i* ∈ *N*, *then if a mixed strategy* (*x*^1^, ⋯, *x*^*n*^)∈*S* *is an ex post weighted equilibrium of MG with the combination* *W* = (*W*^1^, ⋯, *W*^*n*^) *of weight sets, then it is also a corresponding robust weighted Nash equilibrium of MG*.

**Proof.**Let (*x*^1^, ⋯, *x*^*n*^)∈**S** be an *ex post* weighted equilibrium of MG. We will prove this lemma by contradiction, that is, (*x*^1^, ⋯, *x*^*n*^) is not a robust weighted Nash equilibrium. This means that there is *i* ∈ {1, ⋯, *N*} and **u**^*i*^ ∈ **S**_*a*_*i*__, such that

$$\begin{array}{c} \max_{w^{i}\in W^{i}}{\left(w^{i}\right)}^{T}F^{i}\left(x^{-i},x^{i}\right)>\max_{w^{i}\in W^{i}}{\left(w^{i}\right)}^{T}F^{i}\left(x^{-i},u^{i}\right). \end{array}$$ (5)

From the definition of *ex post* weighted equilibrium, ∀*i* ∈ *N*,

$$\begin{array}{c} {\left(w^{i}\right)}^{T}F^{i}\left(x^{-i},u^{i}\right)\geq {\left(w^{i}\right)}^{T}F^{i}\left(x^{-i},x^{i}\right),\;\;\forall w^{i}\in W^{i}. \end{array}$$

This means that

$$\begin{array}{c} \max_{w^{i}\in W^{i}}{\left(w^{i}\right)}^{T}F^{i}\left(x^{-i},x^{i}\right)\leq \max_{w^{i}\in W^{i}}{\left(w^{i}\right)}^{T}F^{i}\left(x^{-i},u^{i}\right), \end{array}$$

which contradicts Eq (3). This contradiction means that (*x*^1^, ⋯, *x*^*n*^)∈**S** is a robust weighted Nash equilibrium of MG with weight combination set *W*.

Because the *ex post* weighted equilibrium concept is too strong, it may not exist for some MG. So we will use the concept of robust weighted equilibrium for MG and in the following section, we will prove that this equilibrium always exists for all MG.

## 3 Existence of robust weighted Nash equilibria in MG

In this section, we first present the existence theorem of robust weighted Nash equilibria in MG with compact and convex weight sets by using Kakutani’s fixed point theorem proposed by Kakutani in [30] which has been extensively used to prove the existence of the quilibrium ([17, 18]). We show that the robust-weighted Nash equilibria of a worst-case weighted multi-objective game are guaranteed to exist even when the weighted set is unbounded. Second, we discuss the conditions for ensuring the existence of robust weighted Nash equilibria. Before continuing to the existence theorem, we first give Kahutani’s fixed point theorem and a relevant definition–*upper-semi continuity*.

**Definition 3.1** *A point-to-set mapping* *ψ*: *S* → 2^*S*^ *is said to be upper semi-continuous if* *y*^*n*^ ∈ *ψ*(*x*^*n*^), *n* = 1, 2, 3, ⋯, $\lim_{n\to \infty }x^{n}=x$, $\lim_{n\to \infty }y^{n}=y$ *imply that* *y* ∈ *ψ*(*x*).

**Theorem 3.2** (Kakutani’s fixed point theorem). Suppose that *S* is a closed, bounded, and convex set in a Euclidean space, and *ψ* is an upper semi-continuous point-to-set mapping from *S* to the family of closed, convex subsets of *S*, then ∃*x* ∈ *S* such that *x* ∈ *ψ*(*x*).

To utilize the above theorem, we first define a suitably constructed correspondence which fixed point is an equilibrium. For this purpose, for any given *x* = (*x*^1^, ⋯, *x*^*n*^)∈*S*, *y* = (*y*^1^, ⋯, *y*^*n*^)∈*S*, and weight sets combination $W=\left(W^{1},\cdots ,W^{n}\right)\subset W_{r}=\left(W_{r}^{1},\cdots ,W_{r}^{n}\right)$, we define

$$\begin{array}{ccc} & & \phi^{W}\left(x\right):=\left\{z\in S|\;z\in arg\min_{y\in S}\rho^{W}\left(x,y\right)\right\}, \end{array}$$

where for any *i* ∈ *N*, the function *ρ*^*W*^: *S* × *S* → *R* is given as follows,

$$\begin{array}{c} \rho^{W}\left(x,y\right):=\sum_{i=1}^{n}\max_{w^{i}\in W^{i}}{\left(w^{i}\right)}^{T}F^{i}\left(x^{-i},y^{i}\right). \end{array}$$

Similar with the proof of Lemma 2.3 given by [2], it is easy to see from the following lemma that the equivalence of the robust weighted Nash equilibrium with the fixed point of the correspondence *ϕ*^*W*^.

**Lemma 3.3** *A given strategy combination* $\bar{x}\in S$ *is a robust weighted Nash equilibrium of MG with* $W=\left(W^{1},\cdots ,W^{n}\right)\subset W_{r}=\left(W_{r}^{1},\cdots ,W_{r}^{n}\right)$ *if and only if* $\bar{x}$ *is a fixed point of the mapping* *ϕ*^*W*^.

**Proof.** If $\bar{x}\in S$ is a robust weighted Nash equilibrium of MG with given $W\subset \tilde{W}$, from Definition 2.5,

$$\begin{array}{c} \max_{w_{i}\in W_{i}}{\left(w_{i}\right)}^{T}F_{i}\left({\bar{x}}_{-i},{\bar{x}}_{i}\right)\leq \max_{w_{i}\in W_{i}}{\left(w_{i}\right)}^{T}F_{i}\left({\bar{x}}_{-i},y_{i}\right),\;\forall y^{i}\in S_{i},\;i=1,\cdots ,m. \end{array}$$

The above inequality implies that

$$\begin{array}{c} \sum_{i=1}^{m}\max_{w_{i}\in W_{i}}{\left(w_{i}\right)}^{T}F_{i}\left({\bar{x}}_{-i},{\bar{x}}_{i}\right)\leq \sum_{i=1}^{m}\max_{w_{i}\in W_{i}}{\left(w_{i}\right)}^{T}F_{i}\left({\bar{x}}_{-i},y_{i}\right),\;\forall y^{i}\in S_{i},\;i=1,\cdots ,m. \end{array}$$

So from the definition of *ρ*_*W*_, $\rho_{W}\left(\bar{x},\bar{x}\right)\leq \rho_{W}\left(\bar{x},y\right)$, ∀*y* ∈ *S*. Therefore $\bar{x}\in \phi_{W}\left(\bar{x}\right)$, i.e., $\bar{x}$ is a fixed point of the mapping *ϕ*_*W*_.

Similarly, for any given $W=\left(W_{1},\cdots ,W_{m}\right)\subset \tilde{W}=\left({\tilde{W}}_{1},\cdots ,{\tilde{W}}_{m}\right)$, if $\bar{x}$ is a fixed point of the mapping *ϕ*_*W*_, we can prove that $\bar{x}\in S$ is also a robust weighted Nash equilibrium of MG with the weight set combination *W*.

The above lemma shows that the existence of the robust weighted Nash equilibria is equivalent to the existence of the fixed points of the mapping *ϕ*^*W*^. So we need to show that the correspondence *ϕ*^*W*^ satisfies the assumptions of Kakutani’s theorem that is to show that for weight sets combination *W*, under some given assumptions about the functions $f_{j}^{i}\left(x^{-i},x^{i}\right)$, ∀*j* = 1, ⋯, *b*_*i*_, *i* ∈ *N*, *ϕ*^*W*^ is an upper semi-continuous point-to-set mapping from *S* to the family of closed, convex subsets of *S*. To reveal that the correspondence *ϕ*^*W*^ meets the assumptions of Kakutani’s theorem, we first need several technical results.

**Lemma 3.4** *Given weight sets combination* *W* ⊂ *W*_*r*_. *If* $f_{j}^{i}\left(\cdot \right)$ *is continuous on* *S* *and for any fixed* *x*^−*i*^, $f_{j}^{i}\left(x^{-i},\cdot \right)$ *is convex on* *S*_*i*_, ∀*j* = 1, ⋯, *b*_*i*_, *i* ∈ *N*, *then we have that*

1. *ρ*^*W*^(⋅, ⋅) is continuous on *S* × *S*;
2. for any fixed *x* ∈ *S*, *ρ*^*W*^(*x*, ⋅) is convex on *S*.

**Proof.** 1. We need to show that for any given *ϵ* > 0, there is a positive constant *δ* such that for any (*x*, *y*)∈*S* × *S* and $(\bar{x},\bar{y})\in S\times S$ if $∥\left(x,y\right)-\left(\bar{x},\bar{y}\right)∥\leq \delta$ then

$$\begin{array}{c} |\rho^{W}\left(x,y\right)-\rho^{W}\left(\bar{x},\bar{y}\right)|\leq \epsilon . \end{array}$$ (6)

It follows from the continuity of $f_{j}^{i}\left(\cdot \right)$ that for any given *ϵ*_*i*_ > 0, there is a positive constant *δ*_*i*_ such that for any (*x*, *y*)∈*S* × *S* and $(\bar{x},\bar{y})\in S\times S$ if $∥\left(x^{-i},y^{i}\right)-\left({\bar{x}}^{-i},{\bar{y}}^{i}\right)∥\leq \delta_{i}$ then

$$\begin{array}{c} ∥F^{i}\left(x^{-i},y^{i}\right)-F^{i}\left({\bar{x}}^{-i},{\bar{y}}^{i}\right)∥\leq \epsilon_{i},\;\forall i\in N. \end{array}$$

The above inequality means that

$$\begin{array}{ccc} & & |\max_{w^{i}\in W^{i}}{\left(w^{i}\right)}^{T}F^{i}\left(x^{-i},y^{i}\right)-\max_{w^{i}\in W^{i}}{\left(w^{i}\right)}^{T}F^{i}\left({\bar{x}}^{-i},{\bar{y}}^{i}\right)|\\ & & \leq \max_{w^{i}\in W^{i}}∥w^{i}∥∥F^{i}\left(x^{-i},y^{i}\right)-F^{i}\left({\bar{x}}^{-i},{\bar{y}}^{i}\right)∥\\ & & =∥F^{i}\left(x^{-i},y^{i}\right)-F^{i}\left({\bar{x}}^{-i},{\bar{y}}^{i}\right)∥\leq \epsilon_{i},\;\forall i\in N. \end{array}$$ (7)

Therefore it follows from Eq (7) and

$$\begin{array}{c} ∥\left(x,y\right)-\left(\bar{x},\bar{y}\right)∥\leq \sum_{i\in N}∥\left(x^{-i},y^{i}\right)-\left({\bar{x}}^{-i},{\bar{y}}^{i}\right)∥ \end{array}$$

that given $\epsilon :=\sum_{i\in N}\epsilon_{i}$, there is $\delta :=\sum_{i\in N}\delta_{i}$ such that $∥\left(x,y\right)-\left(\bar{x},\bar{y}\right)∥\leq \delta$ and Eq (6) holds.

2. For any fixed *x* ∈ *S*, given λ ∈ [0, 1] and $y,\;\bar{y}\in S$, we have that

$$\begin{array}{ccc} & & \rho^{W}\left(x,\lambda y+\left(1-\lambda \right)\bar{y}\right)=\sum_{i=1}^{n}\max_{w^{i}\in W^{i}}{\left(w^{i}\right)}^{T}F^{i}\left(x^{-i},\lambda y^{i}+\left(1-\lambda \right){\bar{y}}^{i}\right)\\ & & \leq \sum_{i=1}^{n}\max_{w^{i}\in W^{i}}{\left(w^{i}\right)}^{T}\left(\lambda F^{i}\left(x^{-i},y^{i}\right)+\left(1-\lambda \right)F^{i}\left(x^{-i},{\bar{y}}^{i}\right)\right)\\ & & \leq \lambda \sum_{i=1}^{n}\max_{w^{i}\in W^{i}}{\left(w^{i}\right)}^{T}F^{i}\left(x^{-i},y^{i}\right)+\left(1-\lambda \right)\sum_{i=1}^{n}\max_{w^{i}\in W^{i}}{\left(w^{i}\right)}^{T}F^{i}\left(x^{-i},{\bar{y}}^{i}\right)\\ & & =\lambda \rho^{W}\left(x,y\right)+\left(1-\lambda \right)\rho^{W}\left(x,\bar{y}\right), \end{array}$$

where the first inequality comes from the convexity of $f_{j}^{i}\left(x^{-i},\cdot \right)$.

The above lemma gives the continuity and convexity for function *ρ*^*W*^ which are two key results for proving the main existence theorem below. The two results are used to prove the upper semi-continuity and convexity of the mapping *ϕ*^*W*^ respectively. We are now going to propose the main result of this section.

**Theorem 3.5** *Suppose that each strategy set* *S*_*i*_ *is a nonempty compact and convex subset of* $R^{a_{i}}$, ∀*i* ∈ *N*. *Then under the conditions of Lemma 3.4, the multi-objective game* *MG* = (*N*, {*S*_*i*_}_*i* ∈ *N*_, {*F*^*i*^}_*i* ∈ *N*_) *has at least one robust weighted Nash equilibrium with* *W* ⊂ *W*_*r*_.

**Proof.**From Lemma 3.3, for the proof of this theorem, it is sufficient to show that the correspondence *ϕ*^*W*^ meets the assumptions of Kakutani’s theorem. To this end, we need to show that *ϕ*^*W*^ is an upper semi-continuous point-to-set mapping from *S* to the family of closed, convex subsets of *S*. We first show that for any given *x* ∈ *S*, *ϕ*^*W*^(*x*) is a closed, convex subsets of *S*.

From the continuity of *ρ*^*W*^(⋅, ⋅) and the existence of minimum of the continuous function on a compact set, we have that $arg\underset{y\in S}{\text{min}}\rho^{W}\left(x,y\right)\neq \emptyset$. Therefore *ϕ*^*W*^(*x*)≠∅, for any *x* ∈ *S*. Note that by definition, *ϕ*^*W*^(*x*)∈*S*, ∀*x* ∈ *S*. Next we will show that *ϕ*^*W*^(⋅) is convex for any *x* ∈ *S*. Suppose that *z*: = (*z*^1^, ⋯, *z*^*n*^), *v*: = (*v*^1^, ⋯, *v*^*n*^)∈*ϕ*(*x*). Then for any λ ∈ [0, 1], we have

$$\begin{array}{c} \rho^{W}\left(x,\lambda z+\left(1-\lambda \right)v\right)\leq \lambda \rho^{W}\left(x,z\right)+\left(1-\lambda \right)\rho^{W}\left(x,v\right)\leq \rho^{W}\left(x,y\right),\;\forall y\in S, \end{array}$$ (8)

where the first inequality comes from the convexity of *ρ*^*W*^(*x*, ⋅) and the second inequality comes from the definition for *z* and *v*. The above inequality and the convexity of *S* mean that *λz* + (1 − λ)*v* ∈ *ϕ*^*W*^(*x*) which implies that *ϕ*^*W*^(⋅) is convex for any *x* ∈ *S*.

Finally, we must show that *ϕ*^*W*^(⋅) is upper semi-continuous correspondence. Suppose that *x*_*n*_ → *x*, *z*_*n*_ → *z*, and *z*_*n*_ ∈ *ϕ*^*W*^(*x*_*n*_). Then we have that

$$\begin{array}{c} \rho^{W}\left(x_{n},z_{n}\right)\leq \rho^{W}\left(x_{n},y\right),\;\forall y\in S. \end{array}$$

Take limit in the above inequality and note that the continuity of *ρ*^*W*^(⋅, ⋅), then we have that

$$\begin{array}{c} \rho^{W}\left(x,z\right)\leq \rho^{W}\left(x,y\right),\;\forall y\in S. \end{array}$$

The above inequality and the compactness of set *S* imply that *z* ∈ *ϕ*^*W*^(*x*). Therefore, we complete the proof that *ϕ*^*W*^ is an upper semi-continuous correspondence and the closedness of the set *ϕ*^*W*^ for any *x* ∈ *S* follows from the upper-semi-continuity of *ϕ*. Therefore, *ϕ*^*W*^ meets the assumptions of Kakutani’s fixed point theorem.

The above theorem presents the existence result for robust weighted Nash equilibrium with compact weight set *W* ⊂ *W*_*r*_. However, in the following corollary we show that even with noncompact weight set there is also at least one robust weighted Nash equilibrium.

**Corollary 3.6** *Under the condition of Theorem 3.5, the multi-objective game* *MG* = (*N*, {*S*_*i*_}_*i* ∈ *N*_, {*F*^*i*^}_*i* ∈ *N*_) *has at least one robust weighted Nash equilibrium with* $W:=\left(W^{1},\cdots ,W^{n}\right)\subset R_{+}^{b_{1}}\times \cdots \times R_{+}^{b_{n}}$ *with* 0 ∉ *W*^*i*^, ∀*i* ∈ *N*.

**Proof.** The proof for this corollary directly follows from Theorem 3.5 and Lemma 2.4 by normalizing the weight set *W* as follows

$$\begin{array}{c} \bar{W}:=\left\{\bar{w}:=\left({\bar{w}}^{1},\cdots ,{\bar{w}}^{n}\right)\left|{\bar{w}}^{i}:=\frac{w^{i}}{∥w^{i}∥},\;w^{i}\in W^{i},\;\forall i\in N\right.\right\}. \end{array}$$

## 4 The computation for robust weighted Nash equilibrium point

Now that the existence of the robust weighted Nash equilibrium point of MG has been presented, our next step is to discuss how to realize such a point. We will show that when the weight sets *W*^*i*^, ∀*i* ∈ *N*, are given as polyhedral region, the function $f_{j}^{i}$ satisfies the assumptions in Lemma 3.4 and furthermore for any fixed *x*^−*i*^, $f_{j}^{i}\left(x^{-i},\cdot \right)$ is continuously differentiable on the compact convex set *S*_*i*_, ∀*j* = 1, ⋯, *b*_*i*_, *i* ∈ *N*, the problem of computing a robust weighted Nash equilibrium point could be cast as the mathematical programming with equilibrium constraints (MPEC) which has been extensively studied and can be solved by the sequential quadratic programming (SQP) method (see [8, 9]).

To this end, we suppose that the uncertain weights can be described by a fixed reference point and a perturbation region around the point, that is the weight set *W*^*i*^ can be given as follows: for any player *i* ∈ *N*, let ${\bar{w}}^{i}\in R^{b_{i}}$ be the reference point of *w*^*i*^ and $C^{i}\in R^{m_{i}\times b_{i}}$ be a coefficient matrix used to construct a perturbation region around ${\bar{w}}^{i}$, then define the perturbation region around ${\bar{w}}^{i}$,

$$\begin{array}{ccc} & & W^{i}=\left\{w^{i}\in R^{b_{i}}|\;w^{i}={\bar{w}}^{i}+{\left(C^{i}\right)}^{T}v^{i},\;v^{i}\in U^{i}\subset R^{m_{i}}\right\},\;\forall i\in N \end{array}$$ (9)

where *U*^*i*^ is the uncertain set which belongs to

$$\begin{array}{c} {\tilde{U}}^{i}=\left\{v^{i}\in R^{m_{i}}|\;{\bar{w}}^{i}+{\left(C^{i}\right)}^{T}v^{i}\geq 0,\;e_{i}^{T}\left({\bar{w}}^{i}+{\left(C^{i}\right)}^{T}v^{i}\right)=1\right\},\;\forall i\in N, \end{array}$$ (10)

here $e_{i}\in R^{b_{i}}$ is the unit vector. Note that the above method for defining uncertain parameters has been widely utilized in robust optimization literature [10, 11]. Define

$$\begin{array}{ccc} & & U^{i}:=\left\{v^{i}\in {\tilde{U}}^{i}|\;A^{i}v^{i}=\gamma^{i},\;B^{i}v^{i}≽c^{i}\right\},\;\forall i\in N \end{array}$$ (11)

where $A^{i}\in R^{l_{i}\times m_{i}}$, $\gamma^{i}\in R^{l_{i}}$, $c^{i}\in R^{k_{i}}$ and $B^{i}\in R^{k_{i}\times m_{i}}$. We now present a reformulation for *RoP*(*W*^*i*^) in Definition 2.5 with *W*^*i*^ as Eqs (9)–(11) and then we show that the computation for a robust weighted Nash equilibrium point of MG is equivalent to solve a mathematical programming problem with equilibrium constraints (MPEC). We note that for designing the computation method, the set *S*_*i*_ is supposed to be nonempty and can be defined as

$$\begin{array}{c} S_{i}=\left\{x^{i}\in R^{a_{i}}|\;x^{i}\geq 0,\;\sum_{j_{i}=1}^{a_{i}}x_{j_{i}}^{i}=1\right\},\;\forall i\in N. \end{array}$$ (12)

**Lemma 4.1** *For any player i, suppose that the weight set* *W*^*i*^ *is defined by Eqs (9)–(11). Then* *RoP*(*W*^*i*^) *is equivalent to the following convex optimization problem*,

$$\begin{array}{ccc} & & \min_{u^{i},z^{i1},z^{i2},z^{i3},z^{i4}}{\left({\bar{w}}^{i}\right)}^{T}z^{i3}+z^{i4}-{\left(\gamma^{i}\right)}^{T}z^{i1}-{\left(c^{i}\right)}^{T}z^{i2}\\ & & s.t.\;\;C^{i}z^{i3}+{\left(A^{i}\right)}^{T}z^{i1}+{\left(B^{i}\right)}^{T}z^{i2}=0\\ & & \;\;\;\;\;\;\;F^{i}\left(x^{-i},u^{i}\right)-z^{i3}-z^{i4}e\leq 0\\ & & \;\;\;\;\;\;z^{i1}\in R^{l_{i}},\;z^{i2}\in R_{+}^{k_{i}},\;z^{i3}\in R^{b_{i}},\;z^{i4}\in R\\ & & \;\;\;\;\;\;u^{i}\in S_{i}. \end{array}$$ (13)

**Proof.** For any player *i*, it is easy to show that *RoP*(*W*^*i*^) can be rewritten as

$$\begin{array}{ccc} & & \min_{u^{i},t}t\\ & & s.t.\;\;\max_{w^{i}\in W^{i}}\;\;{\left(w^{i}\right)}^{T}F^{i}\left(x^{i},u_{i}\right)\leq t,\\ & & \;\;\;\;\;\;u^{i}\in S_{i}. \end{array}$$ (14)

For any given *x* = (*x*^−*i*^, *u*^*i*^), the left hand-side in the first inequality constraint in the above problem is equivalent to

$$\begin{array}{ccc} & & \max_{v^{i}}(C^{i}F^{i}{\left(x^{-i},u^{i})\right)}^{T}v^{i}+{\left({\bar{w}}^{i}\right)}^{T}F^{i}\left(x^{-i},u^{i}\right),\\ & & s.t.\;\;{\bar{w}}^{i}+{\left(C^{i}\right)}^{T}v^{i}\geq 0,\;e_{i}^{T}\left({\bar{w}}^{i}+{\left(C^{i}\right)}^{T}v^{i}\right)=1,\\ & & \;\;\;\;\;\;A^{i}v^{i}=\gamma^{i},\;B^{i}v^{i}\geq c^{i}. \end{array}$$ (15)

The corresponding dual problem is

$$\begin{array}{ccc} & & \min_{z^{i1},z^{i2},z^{i3},z^{i4}}{\left({\bar{w}}^{i}\right)}^{T}z^{i3}+z^{i4}-{\left(\gamma^{i}\right)}^{T}z^{i1}-{\left(c^{i}\right)}^{T}z^{i2},\\ & & s.t.\;\;C^{i}z^{i3}+{\left(A^{i}\right)}^{T}z^{i1}+{\left(B^{i}\right)}^{T}z^{i2}=0\\ & & \;\;\;\;\;\;\;F^{i}\left(x^{-i},u^{i}\right)-z^{i3}-z^{i4}e\leq 0\\ & & \;\;\;\;\;\;z^{i1}\in R^{l_{i}},\;z^{i2}\in R_{+}^{k_{i}},\;z^{i3}\in R^{b_{i}},\;z^{i4}\in R, \end{array}$$ (16)

where “0” and “e” are the zero and unit vectors with appropriate dimension respectively.

Therefore it follows from Eqs (14)–(16) and the strong duality theorem that the assertion of this theorem is true.

**Theorem 4.2** *Suppose that the function* $f_{j}^{i}$ *satisfies the assumptions in Lemma 3.4 and furthermore for any fixed* *x*^−*i*^, $f_{j}^{i}\left(x^{-i},\cdot \right)$ *is continuously differentiable on the compact convex set* *S*_*i*_ *given by Eq (12)*, ∀*j* = 1, ⋯, *b*_*i*_, *i* ∈ *N*, *then the computation for a robust weighted Nash equilibrium point of MG with the weight sets combination* *W* = (*W*^1^, ⋯, *W*^*n*^) *is equivalent to solve a MPEC, here* *W*^*i*^ *is given as Eqs (9)–(11), for any* *i* ∈ *N*.

**Proof.** For any given *x*^−*i*^, define the Lagrangian of Eq (13) as

$$\begin{array}{ccc} & & L^{i}\left(\left(x^{-i},u^{i}\right),\left(z^{i1},z^{i2},z^{i3},z^{i4}\right),\left(\lambda^{i1},\lambda^{i2},\lambda^{i3},\lambda^{i4},\lambda^{i5}\right)\right)\\ & & \;\;\;\;\;\;\;:={\left({\bar{w}}^{i}\right)}^{T}z^{i3}+z^{i4}-{\left(\gamma^{i}\right)}^{T}z^{i1}-{\left(c^{i}\right)}^{T}z^{i2}\\ & & \;\;\;\;\;\;\;+{\left(\lambda^{i1}\right)}^{T}\left(C^{i}z^{i3}+{\left(A^{i}\right)}^{T}z^{i1}+{\left(B^{i}\right)}^{T}z^{i2}\right)\\ & & \;\;\;\;\;\;\;\;+{\left(\lambda^{i2}\right)}^{T}\left(F^{i}\left(x_{-j},u^{i}\right)-z^{i3}-z^{i4}e_{b_{i}}\right)\\ & & \;\;\;\;\;\;\;-{\left(\lambda^{i3}\right)}^{T}z^{i2}-{\left(\lambda^{i4}\right)}^{T}u^{i}\\ & & \;\;\;\;\;\;\;+\lambda^{i5}\left(\sum_{j_{i}=1}^{a_{i}}u_{j_{i}}^{i}-1\right), \end{array}$$ (17)

where (λ^*i*1^, λ^*i*2^, λ^*i*3^, λ^*i*4^, λ^*i*5^) are the Lagrangian multipliers to Eq (13) and *e*_*b*_*i*__ is the *b*_*i*_ dimension unit vector. It follows from the assumption in this theorem we have that the solution to Eq (13) can be obtained by solving its Karush-Kuhn-Tucker (KKT) system,

$$\begin{array}{ccc} & & \;\nabla_{u^{i}}L^{i}=\nabla_{u^{i}}F^{i}\left(x^{-i},u^{i}\right)\lambda^{i2}-\lambda^{i4}+\lambda^{i5}e_{a_{i}}=0;\\ & & \;\;\nabla_{z^{i1}}L^{i}=-\gamma^{i}+A^{i}\lambda^{i1}=0;\\ & & \;\;\nabla_{z^{i2}}L^{i}=-c^{i}+B^{i}\lambda^{i1}-\lambda^{i3}=0;\\ & & \;\;\nabla_{z^{i3}}L^{i}={\bar{w}}^{i}+{\left(C^{j}\right)}^{T}\lambda^{i1}-\lambda^{i2}=0;\\ & & \;\;\nabla_{z^{i4}}L^{i}=1-e_{b_{i}}^{T}\lambda^{i2}=0;\\ & & \;\;\sum_{j_{i}=1}^{a_{i}}u_{j_{i}}^{i}-1=0; \end{array}$$ (18)

$$\begin{array}{ccc} & & F^{i}\left(x^{-i},u^{i}\right)-z^{i3}-z^{i4}e\leq 0,\;\lambda^{i2}\geq 0;\\ & & {\left(\lambda^{i2}\right)}^{T}\left(F^{i}\left(x^{-i},u^{i}\right)-z^{i3}-z^{i4}e_{b_{i}}\right)=0;\\ & & z^{i2}\geq 0,\;u^{i}\geq 0,\;\lambda^{i3}\geq 0,\;\lambda^{i4}\geq 0;\\ & & {\left(\lambda^{i3}\right)}^{T}z^{i2}=0,\;{\left(\lambda^{i4}\right)}^{T}u^{i}=0, \end{array}$$ (19)

here for simplicity we omit the variables in the function *L*^*i*^ and *e*_*a*_*i*__ is the *a*_*i*_ dimension unit vector. Therefore the computation for the robust weighted retailer equilibrium can be obtained by solving the following MPEC,

$$\begin{array}{ccc} & & \min_{x,z,\lambda }\sum_{i=1}^{n}\left(∥\nabla_{x^{i}}L^{i}{∥}^{2}+\sum_{j=1}^{4}∥\nabla_{z^{ij}}L^{i}{∥}^{2}+{\left(\sum_{j_{i}=1}^{a_{i}}x_{j_{i}}^{i}-1\right)}^{2}\right)\\ & & \;\;s.t.\;F\left(x\right)-z^{3}-z^{4}e\leq 0,\;\lambda^{2}\geq 0,\\ & & \;\;\;\;\;\;\;{\left(\lambda^{2}\right)}^{T}\left(F\left(x\right)-z^{3}-z^{4}e\right)=0,\\ & & \;\;\;\;\;\;\;z^{2}\geq 0,\;x\geq 0,\;\lambda^{3}\geq 0,\;\lambda^{4}\geq 0,\\ & & \;\;\;\;\;\;\;{\left(\lambda^{3}\right)}^{T}z^{2}=0,\;{\left(\lambda^{4}\right)}^{T}x=0,\;\forall i\in N, \end{array}$$ (20)

where *F*(*x*) = (*F*^1^(*x*^−1^, *x*^1^), ⋯, *F*^*n*^(*x*^−*n*^, *x*^*n*^)), *e* = (*e*_*b*_1__, ⋯, *e*_*b*_*n*__) *z*: = (*z*^1^, ⋯, *z*^4^), *z*^*j*^: = (*z*^1*j*^, ⋯, *z*^*nj*^), ∀*j* = 1, 2, 3, 4, λ: = (λ^1^, ⋯, λ^5^) and λ^*t*^: = (λ^1*t*^, ⋯, λ^*nt*^), ∀*t* = 1, ⋯, 5.

## 5 An application

Game theory has been extensively studied [31–33] and has been one of the most important tools for analyzing supply chain competition [34–36]. The most of these studies mainly consider the competition there is one objective to be optimized for each player (manufacturer, retailer or consumer) in the supply chain. However in the real-world supply chain, each player often has more than one conflicting objectives to be optimized simultaneously. For example in a supply chain with multiple suppliers who compete in quantities to supply a set of products to satisfy the uncertain consumer demand. Every manufacturer often chooses its supply quantity for each of the products in order to maximize its profit and minimize its cost at the same time by anticipating the order quantities of the retailers and the wholesale prices resulting from the market clearing conditions.

The multiple tiers supply chain has been extensively studied by utilizing game theory. For example, Lau and Lau [35] consider a two-echelon supply chain with one manufacturer and one retailer by game theory. They suppose that a manufacturer wholesales a product to a retailer, who in turn retails it to the consumer under the stochastic demand. Corbett and Karmarkar [36] first consider simultaneous quantity competition at multiple tiers in the supply chain, then Adida and DeMiguel [34] generalize this idea by presenting game models to study a two-echelon supply chain competition where multiple manufacturers who compete in quantities to supply a set of products to multiple risk-averse retailers who compete in quantities to satisfy the uncertain consumer demand.

In this paper, we study the retailers multi-objective competition by applying the worst-case weighted multi-objective game. We consider a supply chain which contains multiple risk-averse retailers who compete in quantities of a set of products to satisfy the uncertain consumer demand. In our model, considering the wholesale price each retailer chooses its wholesale market order quantity for each of the products in order to maximize the mean of its profit and minimize the standard deviation of its profit. Each retailer has more than one objectives to be optimized and the corresponding robust weighted Nash equilibrium can be obtained by utilizing the method provided in the last section.

The rest of this section is organized into the following subsections. Subsection 5.1 introduces the multi-objective game models for describing the retailers’ competition. In the decentralized supply chain, section 5.2 discusses the solution of the robust weighted retailer equilibrium under the finite weights and general polyhedral weight sets assumptions respectively. Section 5.3 describes the reformulation for the solution of the robust weighted retailer equilibrium to a linear complementarity problem (LCP) and MPEC under the finite weights and general polyhedral weight sets assumptions respectively. Section 5.4 details several numerical tests for the above models.

### 5.1 Model description

In this section, we consider to model the retailers’ multi-objective competition in a supply chain which contains *N* risk-averse retailers who compete in quantities of *P* products to satisfy the uncertain consumer demand. In this model, each retailer has two objectives (i.e., one is to maximize its expected utility from retail sales and the other is to minimize its risk expressed as the standard deviation of its profit) by deciding its wholesale market order quantity for each of the *P* products with the given wholesale price.

#### 5.1.1 The retail market demand

Suppose that the retail demand satisfies the following stochastic linear inverse demand function:

$$\begin{array}{c} \hat{p}=\left(\hat{a}-\hat{B}\hat{x}\right)\xi , \end{array}$$

where $\hat{p}={\left(p_{1},\cdots ,p_{N}\right)}^{T}\in R^{NP}$ is the aggregate price vector, *p*_*j*_ is the vector of the *j*th retailer prices with *p*_*j*_ = (*p*_*j*1_, ⋯, *p*_*jP*_), *p*_*jk*_ is the price of the *j*th retailer for the *k*th product, and *ξ* is a positive scalar random variable. The vector $\hat{a}={\left(a_{1},\cdots ,a_{N}\right)}^{T}\in R^{NP}$ with *a*_*j*_ = (*a*_*j*1_, ⋯, *a*_*jP*_) gives the prices that the consumers would be willing to pay if the retail market supply was 0 and *ξ* is a deterministic number. The matrix $\hat{B}\in R^{NP\times NP}$ is the positive semidefinite matrix of inverse demand sensitivities which can be expressed as

$$\begin{array}{c} \left(\begin{array}{cccc} H_{1} & G_{12} & \cdots & G_{1N}\\ G_{21} & H_{2} & \cdots & G_{2N}\\ \vdots & \vdots & \ddots & \vdots \\ G_{N1} & G_{N2} & \cdots & H_{N} \end{array}\right), \end{array}$$

here *H*_*j*_ ∈ *R*^*P*×*P*^, *j* = 1, ⋯, *N*, *G*_*jk*_ ∈ *R*^*P*×*P*^, *j*, *k* = 1, ⋯, *N*. The vector $\hat{x}={\left(x_{1},\cdots ,x_{N}\right)}^{T}\in R^{NP}$ is the aggregate retailer order vector, here *x*_*j*_ is the vector of the *j*th retailer order vector *x*_*j*_ = (*x*_*j*1_, ⋯, *x*_*jP*_).

#### 5.1.2 The retailers’ decision model

The *j*th retailer making his decision by choosing its order quantity by considering two competing objectives one is to maximize its profit and the other is to minimize its risk function $r_{j}\left(x_{j},\hat{p}\right)$, defined as the standard deviation of its profit,

$$\begin{array}{ccc} & & max\;\;-x_{j}^{T}v+x_{j}^{T}\left(a_{j}-{\hat{B}}_{j\cdot }\hat{x}\right)E\left[\xi \right],\\ & & min\;\;r_{j}\left(x_{j},\hat{p}\right),\\ & & s.t.\;\;x_{j}\geq 0, \end{array}$$ (21)

where *v* = (*v*_1_, ⋯, *v*_*P*_) is the vector of wholesale prices and ${\hat{B}}_{j\cdot }=\left(G_{j1},\cdots ,H_{j},\cdots ,G_{jN}\right)$, for *j*th retailer whose first objective is to maximize his mean profit and second objective is to minimize his risk function. One of possible choice for the risk function is standard deviation for the stochastic demand, which can be defined as follows, $r_{j}\left(x_{j},\hat{p}\right):=x_{j}^{T}\left(a_{j}-{\hat{B}}_{j\cdot }\hat{x}\right)\sigma \left(\xi \right)$. In the rest of this paper, we assume that the risk function is defined as the standard deviation of the stochastic demand.

### 5.2 The reformulation for the robust weighted equilibrium

In this section we consider the computation for the robust weighted equilibrium. To this end, we suppose that the central decision-maker has an ambiguous idea about the weights for the objectives in model (21) but he knows that the weights should belong to a set of weights. To cope with this setting, we utilize the robust weighted Nash equilibrium concept introduced in section 2.

To this end, we suppose that the central decision-maker has a closed set of weights $W^{j}\subset W_{r}^{j}:=\left\{w^{j}\in R_{+}^{2}|\;∥w^{j}{∥}_{1}=1\right\}$ for retailer *j*, *j* = 1, ⋯*N*. Then each retailer *j*, *j* = 1, ⋯, *N* aims at solving the following robust optimization,

$$\begin{array}{ccc} & & \min_{x_{j}≽0}\max_{w^{j}\in W^{j}}\;\;{\left(w^{j}\right)}^{T}f^{j}\left(x_{-j},x_{j}\right), \end{array}$$ (22)

where $W^{j}\subset W_{r}^{j}$ and

$$\begin{array}{c} f^{j}\left(x_{-j},x_{j}\right):=\left(f_{1}^{j}\left(x_{-j},x_{j}\right),f_{2}^{j}\left(x_{-j},x_{j}\right)\right):=\left(x_{j}^{T}v-x_{j}^{T}\left(a_{j}-{\hat{B}}_{j\cdot }\hat{x}\right)E\left[\xi \right],r_{j}\left(x_{j},\hat{p}\right)\right). \end{array}$$

To guarantee the existence of the equilibrium for any retailer *j*, we note that the choice of *W*^*j*^ should satisfies that for any *w*^*j*^ ∈ *W*^*j*^ the following inequality holds,

$$\begin{array}{c} w_{2}^{j}\sigma \left(\xi \right)-w_{1}^{j}E\left[\xi \right]<0,\;j=1,\cdots ,N. \end{array}$$ (23)

The above inequality can ensure the strict convexity of (*w*^*j*^)^*T*^ *f*^*j*^(*x*_−*j*_, *x*_*j*_) and then the existence of the robust weighted equilibria can be ensured. For any given close set $W^{j}\subset W_{r}^{j}$ for retailer *j*, *j* = 1, ⋯*N*, it is easy to show that any robust optimization solutions to Eq (22) are the Pareto optimal solution to Eq (21).

Note that when the decision-maker can make a deterministic decision about the weights for each retailer, that is the weight set *W*^*j*^, ∀*j* contain one element. Then at this case our model reduces the game theory model with single objective. However in this paper, we consider that the decision-maker is ambiguous about the choice of the weights and he applies a robust optimization approach to cope with the ambiguity of the weights.

We now discuss the optimal retailer supply schedule and pricing based on the above model with *W*^*j*^, *j* = 1, ⋯, *N* are all given by polytope sets. We consider a simplest situation where the weight sets are taken as a convex hull of a finite number of weights.

#### 5.2.1 The LCP reformulation with finite weights

We first give 4 assumptions which will be used to discuss the uniqueness of the robust weighted retailer equilibrium.

**Assumption 1** We suppose that any retailer *j*, *j* = 1, ⋯, *N* has *r* weights vector $w^{jk}:=\left(w_{1}^{jk},w_{2}^{jk}\right)\in R_{++}^{2}$, *k* = 1, ⋯, *r*, with $w_{1}^{jk}+w_{2}^{jk}=1$.

**Assumption 2** Suppose that for any *k* = 1, ⋯, *r*; *l* = 1, ⋯, *m*; *j* = 1, ⋯, *N*,

$$\begin{array}{c} w_{2}^{jk}\sigma \left(\xi \right)-w_{1}^{jk}E\left[\xi \right]<0,\;w^{jk}\in R_{++}^{2}. \end{array}$$

**Assumption 3** The retailer weights satisfy for *k* = 1, ⋯, *r*, *w*^*jk*^ = *w*^*k*^, for all *j*, and the inverse demand intercepts satisfy *a*_*j*_ = *a* for all *j*, and the matrix of inverse demand sensitivities satisfies *H*_*j*_ = *H* for all *j* and *G*_*j*_1_*j*_2__ = *G* for all *j*_1_ ≠ *j*_2_.

**Assumption 4** The following matrix $\hat{H}$ is positive definite,

$$\begin{array}{ccc} & & \hat{H}:=\left[\begin{array}{c} H_{1}\;\;\;\;0\;\;\;\cdots \;0\\ 0\;\;\;\;H_{2}\;\cdots \;0\\ \vdots \;\;\;\;\;\;\;\vdots \;\;\ddots \;\vdots \\ 0\;\;\;\;0\;\;\cdots \;H_{N} \end{array}\right]. \end{array}$$ (24)

**Theorem 5.1** *Suppose that Assumptions 1, 2 and 4 hold. Then the following conclusions are true*:

1. the robust weighted retailer equilibrium is unique and can be obtained by solving the following linear complementarity problem (LCP):
  $$\begin{array}{c} 0\leq {\hat{q}}_{\tilde{k}}+\left(\hat{B}+\hat{H}\right)\hat{x}⊥\hat{x}\geq 0, \end{array}$$ (25)
  *where for any vectors s and t*, 0 ≤ *s*⊥*t* ≥ 0 *means that* *s* ≥ 0, *t* ≥ 0 *and* *s*^*T*^ *t* = 0, ${\hat{q}}_{\tilde{k}}:=\left(-v/\theta^{1\tilde{k}}-a_{1},\cdots ,-v/\theta^{N\tilde{k}}-a_{N}\right)$, $\theta_{jk}:=\frac{w_{2}^{jk}}{w_{1}^{jk}}\sigma \left(\xi \right)-E\left[\xi \right]$, ∀*j*, *k* and $\tilde{k}:=arg\max_{k=1,\cdots ,r}\theta^{jk}$;
2. *if we further have Assumption 3 holds, then the order quantity of each retailer is the unique solution to the following LCP*:
  $$\begin{array}{c} 0\leq -v/\theta^{\tilde{k}}-a+\left(2H+\left(N-1\right)G\right)x⊥x\geq 0, \end{array}$$ (26)
  where $\tilde{k}:=arg\max_{k=1,\cdots ,r}\theta^{k}$.

**Proof.**1. For any given wholesale prices *v*, the retailer *j* will choose his order by solving the following strictly convex optimization problem,

$$\begin{array}{ccc} & & \min_{x_{j}}\;\;\max_{k=1,\cdots ,r}\theta^{jk}x_{j}^{T}\left(a_{j}-{\hat{B}}_{j\cdot }\hat{x}\right)+x_{j}^{T}v,\\ & & s.t.\;\;\;\;\;\;x_{j}\geq 0, \end{array}$$ (27)

where *θ*^*jk*^, *j* = 1, ⋯, *N*. From the Assumptions 1, 2 and 4, we know this conclusion holds.

2. From the above proof and the Assumptions 1, 2, 3 and 4, this assertion is true.

#### 5.2.2 The MPEC reformulation with general polyhedral weight region

In this section, we will discuss the MPEC reformulation for obtaining the robust weighted retailer equilibrium when the weight sets *W*^*j*^ are given as polytope. Similar with the discussion in section 4, the uncertain weights are described by introducing a fixed reference point and a perturbation region around it. That is for any given retailer *j*, let ${\bar{w}}^{j}\in R^{2}$ be the reference point of *w*^*j*^ and *C*^*j*^ ∈ *R*^*r*_*j*_×2^ be a coefficient matrix used to construct a perturbation region around *w*^*j*^, then define the perturbation region around *w*^*j*^ as follows,

$$\begin{array}{ccc} & & W^{j}=\left\{w^{j}\in R^{2}|\;w^{j}={\bar{w}}^{j}+{\left(C^{j}\right)}^{T}\xi^{j},\;\xi^{j}\in S_{w}^{j}\subset R^{r_{j}}\right\},\;\forall j, \end{array}$$ (28)

where $S_{w}^{j}$ is the uncertain sets which belongs to

$$\begin{array}{c} {\tilde{W}}^{j}=\left\{\xi^{j}\in R^{r_{j}}|\;{\bar{w}}^{j}+{\left(C^{j}\right)}^{T}\xi^{j}\geq 0,\;e_{j}^{T}\left({\bar{w}}^{j}+{\left(C^{j}\right)}^{T}\xi^{j}\right)=1\right\},\;\forall j \end{array}$$ (29)

where *e*_*j*_ is the unit vector with appropriate dimension. Define

$$\begin{array}{ccc} & & S_{w}^{j}:=\left\{\xi^{j}\in {\tilde{W}}^{j}|\;\Lambda_{w}^{j}\xi^{j}=b_{w}^{j},\;\Gamma_{w}^{j}\xi^{j}\geq c_{w}^{j}\right\},\;\forall j \end{array}$$ (30)

where $\Lambda_{w}^{j}\in R^{l_{w}^{j}\times r_{j}}$, $\Gamma_{w}^{j}\in R^{k_{w}^{j}\times r_{j}}$. We note that the choice of weight set should satisfy the inequality Eq (23).

We note that in this subsection, we suppose that the order quantities *x*_*j*_ placed by retailer *j* are bounded above, that is there exists some vector *u*_*j*_ ∈ *R*^*P*^ such that *x*_*j*_ ≼ *u*_*j*_. This assumption together with the existence Theorem 3.5 guarantee that there is at least one robust weighted retailer equilibrium. We first present the reformulations for the decision problem of retailers, that is to give the reformulations for the following robust optimization problems, for any *j* = 1, ⋯, *N*,

$$\begin{array}{ccc} & & \min_{x_{j},\chi_{w}^{j}}\chi_{w}^{j}\\ & & s.t.\;\;\max_{w^{j}\in W^{j}}{\left(w^{j}\right)}^{T}f^{j}\left(x_{-j},x_{j}\right)\leq \chi_{w}^{j},\\ & & \;\;\;\;\;\;\;u_{j}\geq x_{j}\geq 0. \end{array}$$ (31)

For any *j* = 1, ⋯, *N*, from the definitions for *W*^*j*^ and Lemma 4.1, Eq (31) is equivalent to the following optimization problem,

$$\begin{array}{ccc} & & \min_{x_{j},z_{w}^{j1},z_{w}^{j2},z_{w}^{j3},z_{w}^{j4}}{\left({\bar{w}}^{j}\right)}^{T}z_{w}^{j3}+z_{w}^{j4}-{\left(b_{w}^{j}\right)}^{T}z_{w}^{j1}-{\left(c_{w}^{j}\right)}^{T}z_{w}^{j2},\\ & & s.t.\;\;C^{j}z_{w}^{j3}+{\left(\Lambda_{w}^{j}\right)}^{T}z_{w}^{j1}+{\left(\Gamma_{w}^{j}\right)}^{T}z_{w}^{j2}=0\\ & & \;\;\;\;\;\;\;f^{j}\left(x_{-j},x_{j}\right)-z_{w}^{j3}-z_{w}^{j4}e\leq 0\\ & & \;\;\;\;\;\;z_{w}^{j1}\in R^{l_{w}^{j}},\;z_{w}^{j2}\in R_{+}^{k_{w}^{j}},\;z_{w}^{j3}\in R^{2},\;z_{w}^{j4}\in R,\;u_{j}\geq x_{j}\geq 0. \end{array}$$ (32)

The above problem is a convex optimization problem with linear objective function and convex constraints set. If we define the Lagrangian of this problem as

$$\begin{array}{ccc} & & L_{j}\left(\left(x_{-j},x_{j}\right),\left(z_{w}^{j1},z_{w}^{j2},z_{w}^{j3},z_{w}^{j4}\right),\left(\lambda_{w}^{j1},\lambda_{w}^{j2},\lambda_{w}^{j3},\lambda_{w}^{j4},\lambda_{w}^{j5}\right)\right):=\\ & & \;\;\;\;\;\;\;{\left({\bar{w}}^{j}\right)}^{T}z_{w}^{j3}+z_{w}^{j4}-{\left(b_{w}^{j}\right)}^{T}z_{w}^{j1}-{\left(c_{w}^{j}\right)}^{T}z_{w}^{j2}\\ & & \;\;\;\;\;\;\;+{\left(\lambda_{w}^{j1}\right)}^{T}\left(C^{j}z_{w}^{j3}+{\left(\Lambda_{w}^{j}\right)}^{T}z_{w}^{j1}+{\left(\Gamma_{w}^{j}\right)}^{T}z_{w}^{j2}\right)\\ & & \;\;\;\;\;\;\;+{\left(\lambda_{w}^{j2}\right)}^{T}\left(f^{j}\left(x_{-j},x_{j}\right)-z_{w}^{j3}-z_{w}^{j4}e\right)\\ & & \;\;\;\;\;\;-{\left(\lambda_{w}^{j3}\right)}^{T}z_{w}^{j2}-{\left(\lambda_{w}^{j4}\right)}^{T}x_{j}+{\left(\lambda_{w}^{j5}\right)}^{T}\left(x_{j}-u_{j}\right), \end{array}$$ (33)

then we have that the solution to Eq (28) can be obtained by solving its Karush-Kuhn-Tucker (KKT) system, omitting arguments $\left(\left(x_{-j},x_{j}\right),\left(z_{w}^{j1},z_{w}^{j2},z_{w}^{j3},z_{w}^{j4}\right),\left(\lambda_{w}^{j1},\lambda_{w}^{j2},\lambda_{w}^{j3},\lambda_{w}^{j4},\lambda_{w}^{j5}\right)\right)$ in the Lagrangian function,

$$\begin{array}{ccc} & & \nabla_{x_{j}}L_{j}=\nabla_{x_{j}}f^{j}\left(x_{-j},x_{j}\right)\lambda_{w}^{j2}-\lambda_{w}^{j4}+\lambda_{w}^{j5}=0;\\ & & \nabla_{z_{w}^{j1}}L_{j}=-b_{w}^{j}+\Lambda_{w}^{j}\lambda_{w}^{j1}=0;\\ & & \nabla_{z_{w}^{j2}}L_{j}=-c_{w}^{j}+\Gamma_{w}^{j}\lambda_{w}^{j1}-\lambda_{w}^{j3}=0;\\ & & \nabla_{z_{w}^{j3}}L_{j}={\bar{w}}^{j}+{\left(C^{j}\right)}^{T}\lambda_{w}^{j1}-\lambda_{w}^{j2}=0;\\ & & \nabla_{z_{w}^{j4}}L_{j}=1-e^{T}\lambda_{w}^{j2}=0;\\ & & f^{j}\left(x_{-j},x_{j}\right)-z_{w}^{j3}-z_{w}^{j4}e\leq 0,\;\lambda_{w}^{j2}\geq 0;\\ & & {\left(\lambda_{w}^{j2}\right)}^{T}\left(f^{j}\left(x_{-j},x_{j}\right)-z_{w}^{j3}-z_{w}^{j4}e\right)=0;\\ & & z_{w}^{j2}\geq 0,\;x_{j}\geq 0,\;\lambda_{w}^{j3}\geq 0,\;\lambda_{w}^{j4}\geq 0;\\ & & {\left(\lambda_{w}^{j3}\right)}^{T}z_{w}^{j2}=0,\;{\left(\lambda_{w}^{j4}\right)}^{T}x_{j}=0;\\ & & \lambda_{w}^{j5}\geq 0,\;x_{j}-u_{j}\leq 0,\;{\left(\lambda_{w}^{j5}\right)}^{T}\left(x_{j}-u_{j}\right)=0. \end{array}$$ (34)

Therefore from Theorem 4.2 the computation for the robust weighted retailer equilibrium can be obtained by solving the following MPEC,

$$\begin{array}{ccc} & & \min_{\hat{x},z_{w},\lambda_{w}}\sum_{j=1}^{N}\left(∥\nabla_{x_{j}}L_{j}{∥}^{2}+\sum_{i=1}^{4}∥\nabla_{z_{w}^{ji}}L_{j}{∥}^{2}\right)\\ & & \;\;s.t.\;f\left(\hat{x}\right)-z_{w}^{3}-z_{w}^{4}e\leq 0,\;\lambda_{w}^{2}\geq 0,\\ & & \;\;\;\;\;\;\;{\left(\lambda_{w}^{2}\right)}^{T}\left(f\left(\hat{x}\right)-z_{w}^{3}-z_{w}^{4}e\right)=0,\\ & & \;\;\;\;\;\;\;z_{w}^{2}\geq 0,\;\hat{x}\geq 0,\;\lambda_{w}^{3}\geq 0,\;\lambda_{w}^{4}\geq 0,\\ & & \;\;\;\;\;\;\;{\left(\lambda_{w}^{3}\right)}^{T}z_{w}^{2}=0,\;{\left(\lambda_{w}^{4}\right)}^{T}\hat{x}=0,\\ & & \;\;\;\;\;\;\;\lambda_{w}^{5}\geq 0,\;\hat{x}-\hat{u}\leq 0,\;{\left(\lambda_{w}^{5}\right)}^{T}\left(\hat{x}-\hat{u}\right)=0, \end{array}$$ (35)

where $z_{w}:=\left(z_{w}^{1},\cdots ,z_{w}^{4}\right)$, $z_{w}^{i}:=\left(z_{w}^{1i},\cdots ,z_{w}^{Ni}\right)$, *i* = 1, 2, 3, 4, $\lambda_{w}:=\left(\lambda_{w}^{1},\cdots ,\lambda_{w}^{5}\right)$, $\lambda_{w}^{k}:=\left(\lambda_{w}^{1k},\cdots ,\lambda_{w}^{Nk}\right)$, *k* = 1, ⋯, 5 and $\hat{u}:=\left(u_{1},\cdots ,u_{N}\right)$.

### 5.3 Numerical tests

In this section, we detail numerical tests for finding a robust weighted retailer equilibrium by using the numerical solver PATH [37]. We present the numerical results for obtaining the robust weighted retailer equilibrium for the models for the decentralized supply chain with finite weights and general polyhedral weight region respectively. We utilize the data given in [34] but with slight change for our purpose. We suppose that *N* = 2 and *P* = 2, that is there are two retailers and two products in the supply chain. We assume that E[ξ]=1 and *σ*(*ξ*) = 0.5, that is the mean and standard deviation of the stochastic variable *ξ* are 1 and 0.5 respectively. We give the following assumption for the parameters in the supply chain,

$$\begin{array}{ccc} & & a_{j}:=\left[\begin{array}{c} 1\\ 1 \end{array}\right],\;j=1,2;\;v:=\left[\begin{array}{c} 0.5\\ 0.5 \end{array}\right]; \end{array}$$ (36)

$$\begin{array}{c} H_{j}=G_{jk}:=\left[\begin{array}{c} 1\;\;\;\;0.5\\ 0.5\;\;\;\;1 \end{array}\right],\;j=1,2. \end{array}$$ (37)

We first use the weighted approach given by Wang [2] and suppose that the retailers allow three possible scale indices for the two objective functions as $f_{1}^{1}/f_{2}^{1}=0.7$, 1 or 1.5, $f_{1}^{2}/f_{2}^{2}=0.8$, 1.28 or 1.69. Then, we assume that the retailer *j* is unsure about the relative importance of $f_{1}^{j}$ and $f_{2}^{j}$, for any *j* = 1, 2. We further assume that there are finite weights to quantify the relative importance for the two objective functions listed as above three possible scale indices. Then we generalize the finite weights to the weight set give by polytope. The numerical results are reported in Table 1 where the equilibria in rows 1–9 of Table 1 are the weighted Nash equilibria depending on the corresponding weights which is given by Wang [2]. Except the equilibria in rows 1 and 5 in Table 1, there are different equilibria for different choice of the weights. The robust weighted equilibrium is computed by using the weight region generated as the convex hull of the nine weight vectors. It is easy to see that the robust weighted approach presents a unique equilibrium which mitigates the conflict with different retailer equilibria for different choice of the weights in rows 1–4 and 6–9 of Table 1. Therefore, the robust weighted approach is more robust than the weighted approach given by Wang [2].

**Table 1:** Numerical test results.

$f_{1}^{1}/f_{2}^{1}$	$f_{1}^{2}/f_{2}^{2}$	Equilibrium
0.7	0.8	(0, 0, 0, 0)
0.7	1.28	(0, 0, 0.06, 0.06)
0.7	1.69	(0, 0, 0.097, 0.097)
1	1.28	(0, 0, 0.06, 0.06)
1	0.8	(0, 0, 0, 0)
1	1.69	(0, 0, 0.097, 0.097)
1.5	1.69	(0.083, 0.083, 0.097, 0.097)
1.5	1.28	(0.083, 0.083, 0.06, 0.06)
1.5	0.8	(0.083, 0.083, 0, 0)
Worst-case weighted approach		(0, 0, 0, 0)

For the general polytope weight region, we suppose the reference point ${\bar{w}}^{j}$ and matrix *C*^*j*^ are defined as ${\bar{w}}^{1}={\bar{w}}^{2}:=\left(1/2,1/2\right)$, and

$$\begin{array}{ccc} & & C^{1}=C^{2}:=\left[\begin{array}{c} 1\;\;\;\;2\\ 1\;\;\;\;2 \end{array}\right]. \end{array}$$ (38)

The perturbation subset is defined as

$$\begin{array}{ccc} & & S_{w}^{j}:=\left\{\xi^{j}\in R^{2}|\;e^{T}\xi^{j}\leq 1\right\},\;j=1,2. \end{array}$$ (39)

The upper bound $\hat{u}:=\left(3,3,3,3\right)\in R^{4}$. With the above data the robust weighted retailer equilibrium with general polytope is (0, 0, 0, 0) which is same with the robust weighted retailer equilibrium generated in the above with finite weights. This result also shows that the worst-case weighted approach is robust.

## 6 Conclusions

In this paper, we introduced a worst-case weighted approach to the multi-objective games. The existence theorem is proved even when the weight sets are unbounded. The computation of the robust weighted equilibrium point, with the polyhedral weight sets, can be equivalently transformed into solving a MPEC. As an application, the new approach is utilized to analyze the supply chain multi-objective competition problem. In this paper, for computation of robust weighted equilibrium point, we consider only that the weight sets are given as polytope without distributional information, however, in many applications, the distributional information is available. So for further study, we will consider to inject distributional information to our model. The further applications to other situations will also be discussed in future.
